# Supplementary material for: A Novel Approach for Investigating Upper Airway Hyperresponsiveness Using Micro-CT in Eosinophilic Upper Airway Inflammation such as Allergic Rhinitis Model
Source: Biomolecules. 2019 Jun 27;9(7):252. doi: 10.3390/biom9070252 (PMC6681309; doi:10.3390/biom9070252)
Supplement: Supplementary file 1 [file biomolecules-09-00252-s001.pdf]

ONLINE SUPPLEMENT

SUPPLEMENTARY FIGURE LEGENDS

Figure S1 Protocol of OVA-induced AR and histology

A indicates protocol schema for OVA-induced AR. **B** shows H&E stain (upper panel) and immunofluorescence images (lower panel). White arrows indicate eosinophil (stained by red).

Figure S2 Analysis of swelling of nasal mucosa using micro-CT images in coronal axis

A and **B** show dose-dependent and kinetics analyses, respectively. Mucosa index was analyzed 15 min after PBS or 0.5–2.0 mg/mL MCh treatment (**A**) or 0, 15, 60, and 120 min after PBS or 1 mg/mL MCh treatment (**B**). Data are expressed as means ± SEMs (n = 3–6 for each group). *Significantly different from PBS-treated mice ($P < 0.05$)

Figure S3 The effect on mucosa index by OVA treatment

Mucosa index was analyzed 15 min after PBS or 1% OVA treatment in healthy control and OVA-induced allergic rhinitis group (n = 3 for each group). Data are expressed as means ± SEMs (n = 3 in each group). n.s.: not significant

Figure S4 Schema of relationship between measurement system and airway hyperresponsiveness in allergic rhinitis and asthma

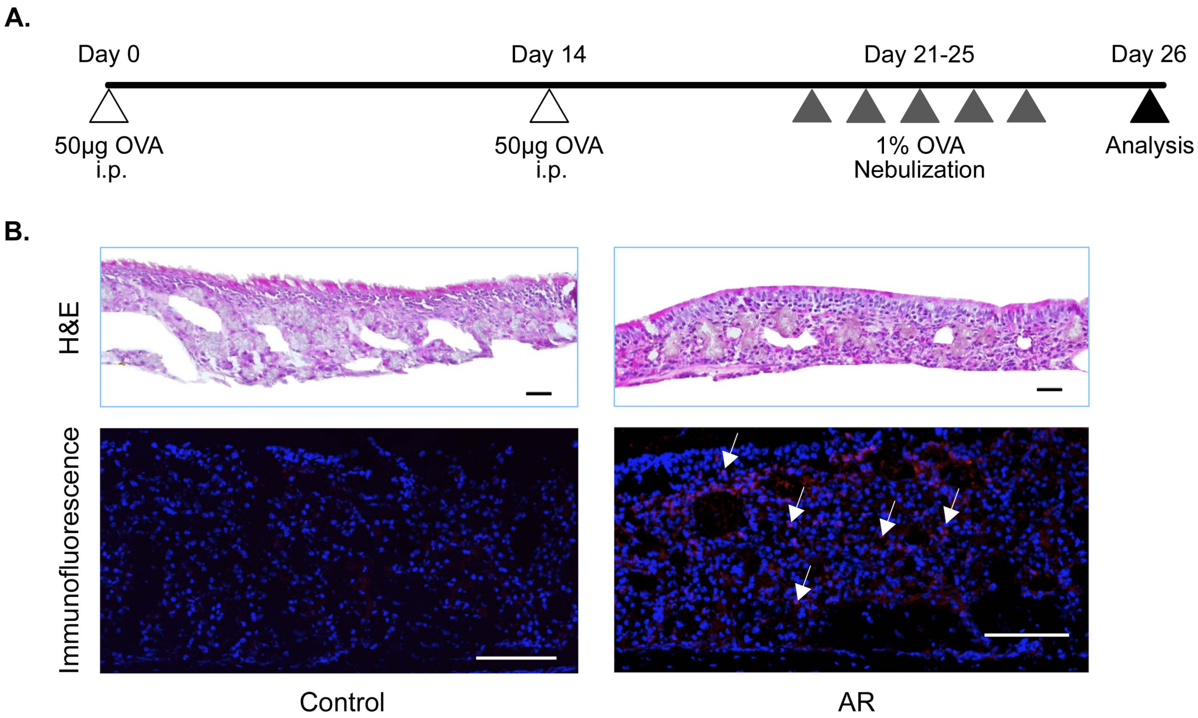

Fig.S1 Bui et al

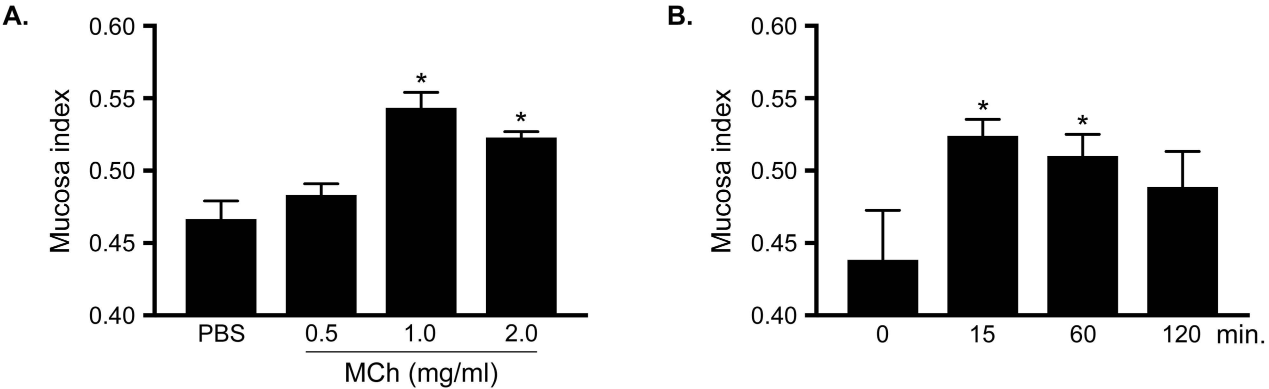

Fig.S2 Bui et al

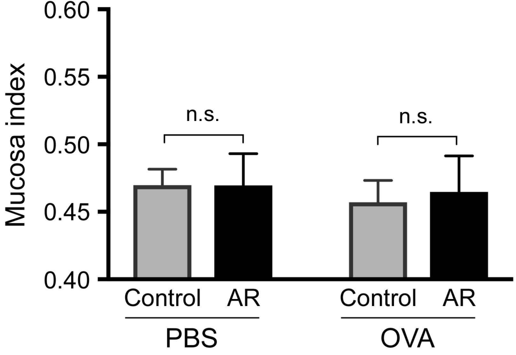

Fig.S3 Bui et al

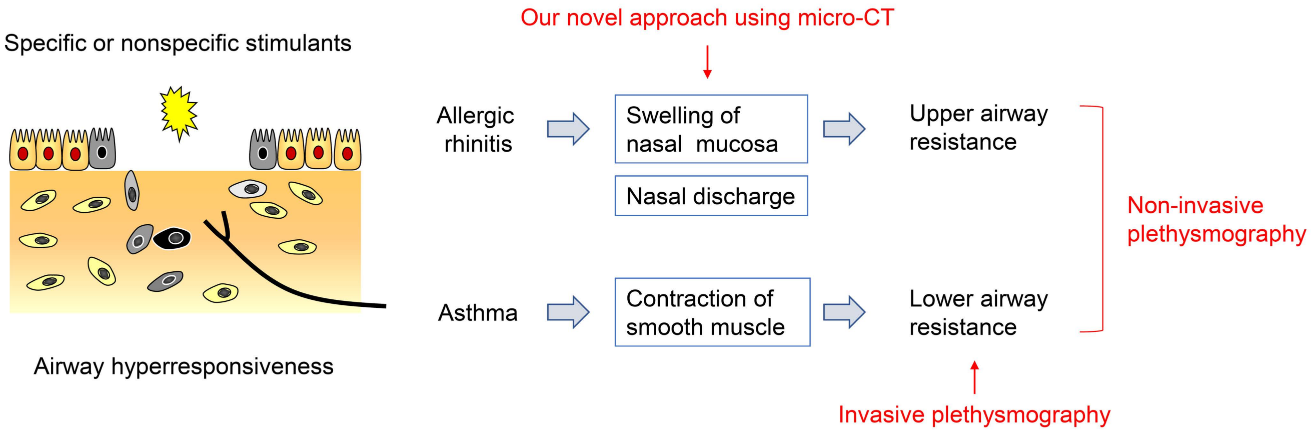

Fig.S4 Bui et al
